# Structural and biochemical insights into PsEst3, a new GHSR-type esterase obtained from *Paenibacillus* sp. R4

**DOI:** 10.1107/S2052252523001562

**Published:** 2023-02-28

**Authors:** Jonghyeon Son, Woong Choi, Hyun Kim, Minseo Kim, Jun Hyuck Lee, Seung Chul Shin, Han-Woo Kim

**Affiliations:** a Research Unit of Cryogenic Novel Material, Korea Polar Research Institute, Incheon 21990, Republic of Korea; b New Drug Development Center, Daegu–Gyeongbuk Medical Innovation Foundation, Daegu 41061, Republic of Korea; c Department of Polar Sciences, University of Science and Technology, Incheon 21990, Republic of Korea; d Division of Life Sciences, Korea Polar Research Institute, Incheon 21990, Republic of Korea; University of Michigan, USA

**Keywords:** X-ray crystallography, protein structure, structure–function relationship, esterases, ligand selectivity, protein engineering, structure determination, enzyme mechanisms, PsEst3, *Paenibacillus* sp. R4

## Abstract

PsEst3, a psychrophilic esterase obtained from *Paenibacillus* sp. R4, which was isolated from the permafrost of Alaska, exhibits relatively high activity at low temperatures. Here, crystal structures of PsEst3 complexed with various ligands were generated and studied at atomic resolution, and biochemical studies were performed to analyze the structure–function relationship of PsEst3. Certain unique characteristics of PsEst3 distinct from those of other classes of lipases/esterases were identified. Firstly, PsEst3 contains a conserved GHSRA/G pentapeptide sequence in the G*x*S*x*G motif around the nucleophilic serine. Additionally, it contains a conserved HGFR/K consensus sequence in the oxyanion hole, which is distinct from that in other lipase/esterase families, as well as a specific domain composition (for example a helix–turn–helix motif) and a degenerative lid domain that exposes the active site to the solvent. Secondly, the electrostatic potential of the active site in PsEst3 is positive, which may cause unintended binding of negatively charged chemicals in the active site. Thirdly, the last residue of the oxyanion hole-forming sequence, Arg44, separates the active site from the solvent by sealing the acyl-binding pocket, suggesting that PsEst3 is an enzyme that is customized to sense an unidentified substrate that is distinct from those of classical lipases/esterases. Collectively, this evidence strongly suggests that PsEst3 belongs to a distinct family of esterases.

## Abstract 2

Crystal structures of PsEst3 complexed with various ligands and its biochemical characterization indicate the emergence of a new GHSR-type lipase/esterase and reveal the relationship between its structure and function.

## 1. Introduction

Esterases (EC 3.1.1.1) and lipases (EC 3.1.1.3), classified as lipases/esterases, catalyze the hydrolysis of various types of substrates, including carboxylic esters, aryl esters and acylglycerols, to produce an acid and an alcohol. These enzymes are widely distributed in animals, plants and microorganisms, and play important roles in lipid metabolism (Lian *et al.*, 2018 ▸) and drug metabolism (Laizure *et al.*, 2013 ▸). Research in recent decades has revealed several characteristics of lipases/esterases. Firstly, many lipases can utilize a broad range of substrates, which increases their spectrum of availability (Li *et al.*, 2008 ▸). Secondly, as chirality is an important factor in the efficacy of many drugs, the stereoselectivity of lipases/esterases is also highly useful. Thirdly, lipases/esterases are stable in organic solvents (Sztajer *et al.*, 1992 ▸). Owing to these characteristics, lipases/esterases have been used as biocatalysts in various industries, including the pharmaceutical industry, in food modification and in detergent production (Guglielmetti *et al.*, 2008 ▸; Bornscheuer, 2002 ▸; Houde *et al.*, 2004 ▸; Panda & Gowrishankar, 2005 ▸).

The structural characteristics of lipases/esterases have been well defined by crystallographic studies (Schrag *et al.*, 1991 ▸; Grochulski *et al.*, 1993 ▸). The structure of lipases/esterases comprises a central backbone formed of β-sheets surrounded by several α-helices, known as an α/β hydrolase fold (Ollis *et al.*, 1992 ▸). Based on the backbone structure, various lipases/esterases harbor common motifs associated with their cleavage mechanisms. For example, catalytic triads that are composed of Ser–Asp/Glu–His and the oxyanion hole, which is a pocket motif responsible for stabilization of the transition state, are located within the loop region between the α-helix and β-strand, forming the active site. However, carboxyl esterases can be distinguished from lipases as they lack interfacial activation due to the absence of the mobile lid domain that is found in lipases (Bordes *et al.*, 2010 ▸). Thus, knowledge of the structure–function relationship of esterases/lipases facilitates their utilization.

Psychrophiles are found in areas with temperatures of <0°C. The most interesting adaptation strategy in these organisms involves cold-active enzymes that exhibit relatively high catalytic activity in low-temperature environments, providing an enormous advantage in terms of saving energy (Gerday *et al.*, 2000 ▸). Together with their genetic variance and their convenient culture conditions, the potential applications of psychrophilic microorganisms in industry are considerable. Protein engineering has proven to be an invaluable tool in the optimization of catalytic efficiency, thermostability and stereoselectivity (Bartsch *et al.*, 2008 ▸). Structural studies on lipases/esterases provide fundamental information for protein engineering. In particular, genome sequencing of *Paeni­bacillus* sp. R4, a species inhabiting the permafrost of Alaska, identified a putative esterase gene (Shin *et al.*, 2020 ▸). Hence, in the current study, we characterized an esterase obtained from *Paenibacillus* sp. R4 (PsEst3) and generated a series of crystal structures at 1.80 Å resolution, providing novel information to facilitate its industrial modification.

## 2. Materials and methods

### 2.1. Sequence analysis

The UniProt database was used to search for homologs of PsEst3. Six homologs with a pentapeptide sequence identical to that of PsEst3 and two with a minor difference in the pentapeptide sequence were selected and aligned. To construct the PsEst3 phylogenetic tree, previously reported sequences from various lipase families (Arpigny & Jaeger, 1999 ▸) were aligned with the four homologs used as input in the multiple sequence alignment and analyzed using the neighbor-joining method (Saitou & Nei, 1987 ▸) with *MEGA X* (Kumar *et al.*, 2018 ▸). The resolved 3D structure of PsEst3 was then used as input for the *DALI* server (Holm, 2020 ▸) to search for similar families of lipases. The sequences and secondary structures of three lipases obtained by the *DALI* server search were aligned and visualized using *ClustalW* and *ESPript* 3.0 (Robert & Gouet, 2014 ▸).

### 2.2. Expression and purification of PsEst3

The expression and purification methods used here were as described previously (Kim *et al.*, 2018 ▸). Briefly, pET-22b encoding PsEst3 was transformed into *Escherichia coli* BL21(DE3)/pPsyGroELS (Kim *et al.*, 2015 ▸). The clone was cultured in Luria–Bertani (LB) broth at 37°C until the OD_600_ reached ∼0.6–0.8. Expression of PsEst3 was induced by the addition of 0.1 m*M* isopropyl β-d-1-thiogalactopyranoside and incubation for two days at 15–18°C. The cells were harvested at 4°C by centrifugation (6000*g*) and the pellet was stored at −80°C until further purification.

The pellet was resuspended in buffer *A* (20 m*M* Tris–HCl pH 8.0, 200 m*M* NaCl) and disrupted via sonication. Cell debris was pelleted by centrifugation (20 000*g*) and the resulting supernatant was purified using an Ni–NTA column (HisTrap HP, GE Healthcare). After washing the column with buffer *B* (20 m*M* Tris–HCl pH 8.0, 500 m*M* NaCl, 20 m*M* imidazole), a linear imidazole gradient (0–500 m*M*) was used to elute PsEst3. The purity was assessed via sodium dodecyl sulfate–polyacrylamide gel electrophoresis (SDS–PAGE) and PsEst3 fractions were pooled and concentrated using Amicon Ultra centrifugal filters (Merck Millipore) for size-exclusion chromatography. The concentrated protein solution was further purified using a HiLoad 16/600 Superdex 200 pg column (GE Healthcare) which had been pre-equilibrated with buffer *A*. Fractions containing PsEst3 were identified using SDS–PAGE and concentrated to 30 mg ml^−1^. The concentrated PsEst3 solution was stored at −80°C.

### 2.3. Site-directed mutagenesis and expression

Plasmids encoding variants of PsEst3 (Ser128 and Arg44 variants) were produced by PCR. The primer pairs for site-directed mutagenesis were designed as described previously (Liu & Naismith, 2008 ▸). The PsEst3 plasmid was used as a template and amplified using the designed primer pairs with KOD DNA polymerase (Toyobo Co. Ltd, Osaka, Japan). The amplified DNA was incubated with DpnI (Takara Bio Inc., Kusatsu, Japan) to remove the template plasmid. The resulting DNA was transformed into *E. coli* DH5α cells and extracted for sequencing. Appropriate colonies were picked based on the sequencing results. Similar expression and purification methods as for wild-type PsEst3 were used.

### 2.4. Crystallization

The initial crystallization of PsEst3 was performed by the sitting-drop vapor-diffusion method using various screening kits. Briefly, 0.5 µl protein solution was mixed with an equal volume of reservoir solution and then incubated at 23°C. Several initial cube-shaped crystals appeared within two days, and two conditions were selected to improve the quality of the crystals. Optimization of the crystals was performed using both the hanging-drop vapor-diffusion and sitting-drop vapor-diffusion methods. Crystals suitable for diffraction were generated using conditions *A* (0.1 *M* Tris pH 7.5, 2.0 *M* ammonium sulfate) and *B* (1.1 *M* malonic acid, 0.15 *M* ammonium citrate tribasic, 0.072 *M* succinic acid, 0.18 *M* dl-malic acid, 0.24 *M* sodium acetate, 0.3 *M* sodium formate, 0.096 *M* ammonium tartrate dibasic pH 7.0).

### 2.5. Diffraction data collection and structure determination

Diffraction data from the two types of crystals were collected on BL-5C at the Pohang Light Source, Pohang, Republic of Korea. The overall strategy of data collection and data processing for the crystals produced using condition *A* was as described previously (Kim *et al.*, 2018 ▸). Data for phenylmethylsulfonyl fluoride (PMSF)-bound PsEst3 were obtained by soaking crystals produced using condition *A* with PMSF powder, since PMSF is not soluble in water. Following 6 h of soaking with PMSF, the crystals were moved to a cryoprotectant drop containing 30% glycerol. The crystals produced using condition *B* were moved into a 25% ethylene glycol drop for cryoprotection and quickly mounted for diffraction. A total of 360 diffraction images were collected at a wavelength of 0.97942 Å with an oscillation angle of 1°; however, only 200 images were used in scaling to exclude radiation-damaged images. Indexing, integration and scaling of the diffraction data were performed using *HKL*-2000 (Otwinowski & Minor, 1997 ▸). The phase was solved by molecular replacement using the *BALBES* system with the stucture of dienelactone hydrolase from *Pseudomonas putida* (PDB entry 1zj4; Kim *et al.*, 2005 ▸) as a model, and the residues specific to PsEst3 were manually constructed using *Coot* (Emsley *et al.*, 2010 ▸). Refinement was conducted using the refinement module of *Phenix* (Afonine *et al.*, 2012 ▸). A detailed summary of data collection and structure refinement is provided in Table 1 ▸.

### 2.6. Determination of the preferred fatty-acid chain length

To measure the activity of PsEst3 towards bulky aromatic alcohol moieties and aliphatic chains, fluorescein dibutyrate (C4), fluorescein dioctanoate (C8) and fluorescein dilaurate (C12) were used as substrates. The fluorescein substrates were prepared in 2-ethoxyethanol at a concentration of 100 µ*M*. The 50 µl reaction mixture consisted of 0.1 µ*M* fluorescein derivative, PsEst3 (1.25 µg was used to measure activity) in 20 m*M* Tris–HCl buffer pH 8.0, 150 m*M* NaCl, 10% 2-ethoxyethanol and 0.005%(*w*/*v*) CHAPS and was directly prepared in a qPCR microtube. Fluorescein signal (SYBR Green filter; λ_ex_ = 492 nm, λ_em_ = 512 nm) was measured at 30°C for 10 min using a Qiagen Rotor-Gene Q 2plex qPCR cycler (Qiagen, Hilden, Germany). The fluorescein standard curve was obtained in the 0.01–0.1 µ*M* range using the same buffer as used for enzymatic measurements (slope = 176.68, intercept = −0.403, *R* ^2^ = 0.9971). All measurements were conducted in triplicate and were corrected for non-enzymatic hydrolysis.

Substrate specificity was investigated using the following commercial *p*-nitrophenyl (*p*NP) esters: acetate, butyrate, hexanoate, octanoate and decanoate. All *p*NP derivatives were dissolved in dimethyl sulfoxide to 2 m*M*. The 80 µl reaction mixture consisted of 50 µg PsEst3 and 250 µ*M* *p*NP ester in 0.1 *M* Tris–HCl pH 8.0 buffer which contained 0.1%(*w*/*v*) gum arabic and 1%(*w*/*v*) CHAPS to minimize autohydrolysis. The PsEst3 was pre-incubated at 30°C for 2 min before the *p*NP ester was added. The reaction was evaluated at 405 nm for 20 min (every 10 s) in a Multiskan GO microplate reader (Thermo Fisher Scientific, Waltham, Massachusetts, USA). Assays were performed in triplicate and the values were normalized for non-enzymatic hydrolysis. The standard curve of the *p*NP derivatives was obtained in the 0–0.5 m*M* range using the same buffer as used for the enzymatic assay (slope = 4.568, intercept = 0.006109, *R* ^2^ = 0.9993).

### 2.7. Effects of varying the pH value and solvent

Various reaction conditions were evaluated for the optimization of PsEst3 activity. While the overall reaction conditions have been described above, the activity was measured at 25°C. The reactions were performed in the pH range 3.5–9.5. Sodium acetate, Tris and glycine buffers were used in the pH ranges 3.5–6.5 and 7.5–8.5 and at pH 9.5, respectively. Reactions conducted above pH 10 were also tested; however, meaningful data were not obtained owing to autohydrolysis of *p*NP-C2. The effects of various solvents including methanol, ethanol, 2-propanol, acetonitrile and dimethyl sulfoxide were also investigated by the addition of these solvents to up to 10% of the total reaction volume.

### 2.8. Substrate-competition assay

A substrate-competition assay was performed to evaluate changes in the *p*NP-C2 hydrolysis activity of PsEst3 following the addition of another substrate. Feruloyl acid, cinnamoyl acid, vanillic acid, malonate, maleic acid and PMSF were individually added to the reaction mixture at concentrations of up to 1.2 m*M*.

## 3. Results and discussion

### 3.1. Functional annotation of genome sequences

The genome of the Arctic bacterium *Paenibacillus* sp. R4 was functionally characterized using the Kyoto Encyclopedia of Genes and Genomes (KEGG; https://www.genome.jp/kegg/) to investigate the functional role of PsEst3 in metabolic pathways. However, a relationship between neighboring genes and PsEst3 was not observed. Moreover, a KEGG number was not assigned to certain neighboring genes by the *KEGG Automatic Annotation Server* (*KAA*S; https://www.genome.jp/kegg/kaas/). Consequently, we were unable to annotate PsEst3 based on genome information.

### 3.2. Crystallographic parameters and model quality

The crystal structure of PsEst3 belonged to the cubic space group *P*4_1_32, with unit-cell parameters *a* = *b* = *c* = 144 Å, α = β = γ = 90°. One molecule was observed in the asymmetric unit, with a solvent content of 71% and a Matthews coefficient of 4.3 Å^3^ Da^−1^. A total of 255 residues (amino acids 4–258), including a partial 6×His tag, were built and refined in the final model, for which the *R*_work_ and *R*_free_ were 0.20 and 0.22, respectively (Table 1 ▸). Four structures were obtained: a sulfate-bound form, the wild type bound to PMSF, a malonate-bound form of the S128A mutant and an apo form of the S128A mutant. These structures were obtained from crystals that were obtained under different conditions but belonged to the same space group with similar unit-cell parameters.

### 3.3. Overall structure of PsEst3

**Figure 1:**
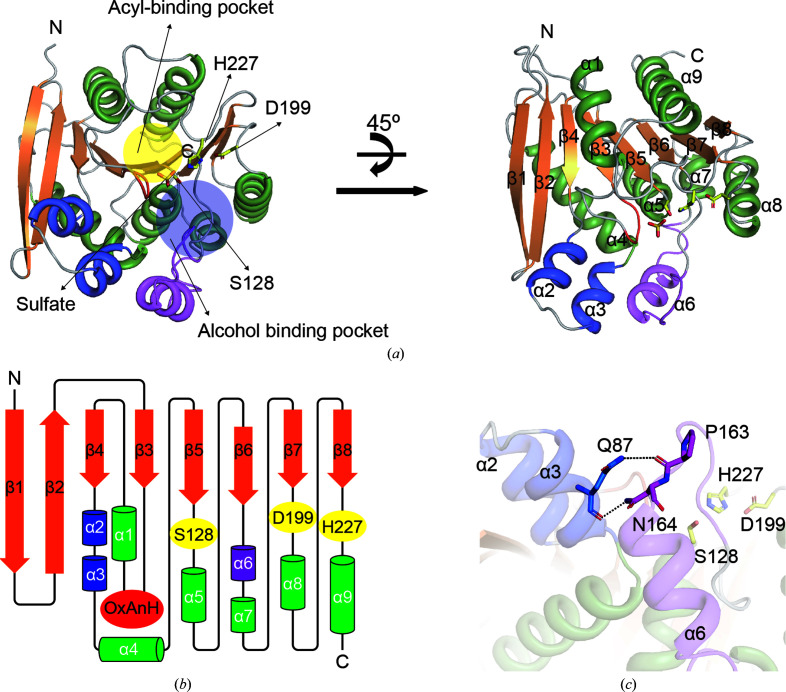
Overall structure of PsEst3. Cartoon representation (*a*) and secondary-structure topology (*b*) of PsEst3. α-Helices and β-sheets are represented as coils and arrows, respectively. A sulfate ion and the catalytic triad (Ser128, Asp199 and His227) are represented as yellow sticks. The tetrapeptide for an oxyanion hole (red) and a PsEst3-specific region (blue and magenta) are highlighted. (*c*) Residues participating in the inter-motif interaction between α3 and α6 of PsEst3 are shown as stick models.

The overall fold of PsEst3 was similar to those of other esterases in the α/β hydrolase family and comprised a central β-sheet surrounded by nine α-helices [Figs. 1 ▸(*a*) and 1 ▸(*b*)]. As eight β-strands were stacked in a helical manner, β1 was nearly perpendicular to β8. Broadly, the α-helices could be divided into two sets of bundles based on their relative orientation to the central β-strand. At the center containing the nucleophile Ser128 two α-helices (α1 and α9) formed the upper side of the central backbone, and several hydrophobic residues, including His41, Arg44, Trp49, Phe51, Trp52 and His127, formed the acyl-binding pocket [Supplementary Fig. S1(*a*)]. In contrast, Phe43, Arg129, Ala158, Ala159, Ala160, Ile167 and Val171 and five α-helices (α4, α5, α6, α7 and α8) located on the opposite side contributed to the formation of an incomplete alcohol-binding pocket [Supplementary Fig. S1(*b*)].

The lid domain, which is a unique feature of lipases that distinguishes them from esterases, participates in interfacial activation. The characteristic lipase from *Candida rugosa* (CRL), which shows dynamic conformational changes between open and closed states, take advantage of the interaction through an anchorage motif that corresponds to the counterpart of α6 observed in PsEst3 [Supplementary Figs. S1(*c*)–S1(*e*)] (Grochulski *et al.*, 1993 ▸). The residues of the anchorage motif in CRL lipase mutually contribute towards stabilization and conformational changes of the lid domain. However, PsEst3 did not contain the counterpart of the lid domain inserted between β1 and β2 of CRL, resulting in the exposure of Ser128 to the solvent. However, despite the absence of a lid domain, a degenerative anchorage domain, α6 in PsEst3, was present. The unique helix–turn–helix motif (α2 and α3; hereafter referred to as the HtH motif) of PsEst3, located in the vicinity of β2, appeared to act as a new anchor for α6, forming inter-motif hydrogen bonds between Gln87, Pro163 and Asn164 [Fig. 1 ▸(*c*)]. Therefore, the composition and arrangement of the secondary structure of PsEst3 exhibited simplification and customization for the catalytic reaction.

**Figure 2:**
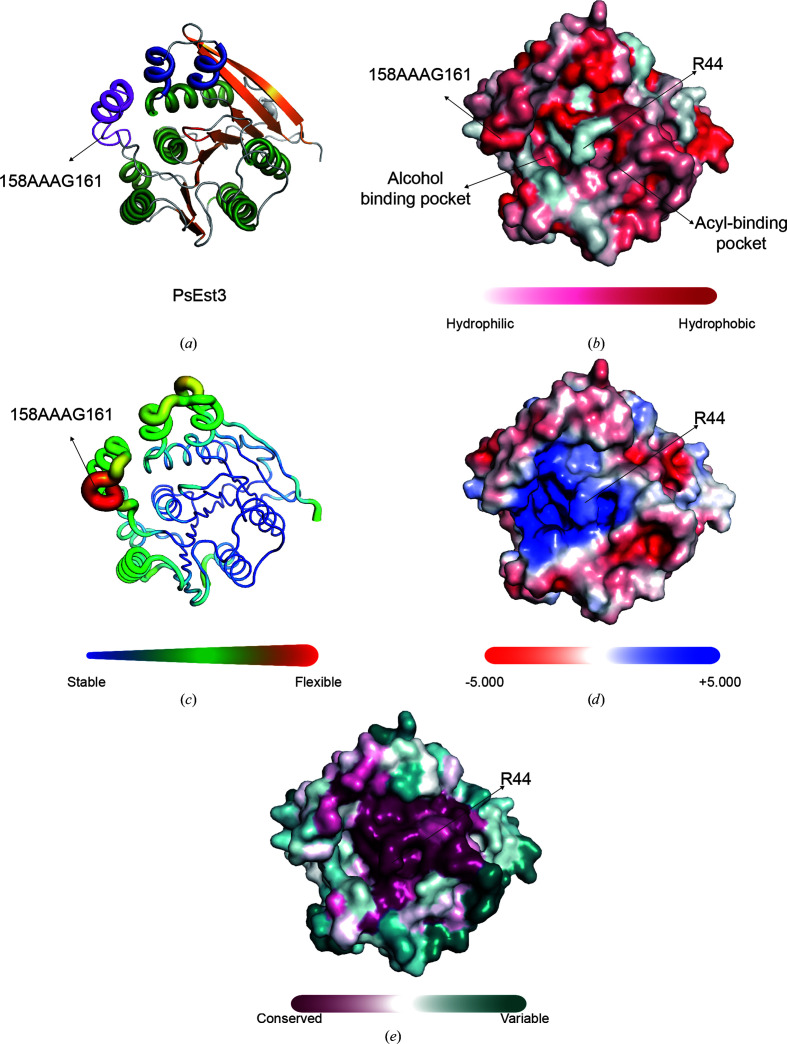
Analysis of the surface characteristics of PsEst3. (*a*) Cartoon representation of the malonate-bound structure of PsEst3. The overall color scheme is identical to that in Fig. 1 ▸(*a*). (*b*) Hydrophobicity of the PsEst3 surface. (*c*) *B*-factor distribution of PsEst3. (*d*) Electrostatic potential of the PsEst3 surface. (*e*) The degree of conservation of PsEst3 residues is represented as a surface model. The degree of conservation was calculated with 250 homologs based on *BLAST* results using the UniProt database. The color gradient of the degree of conservation progresses from magenta to blue–green.

The surface characteristics of PsEst3 were also analyzed (Fig. 2 ▸). The overall solvent-accessible and buried areas of PsEst3 were 22 680 and 1740 Å^2^, respectively. The alcohol-binding pocket was exposed to the solvent, whereas the acyl-binding pocket was partially covered by Arg44 [Fig. 2 ▸(*b*)]. Although the 158-AAAG-161 loop located between β6 and α6 was exposed to solvent, it was composed of hydrophobic residues and exhibited a high *B*-factor distribution [Figs. 2 ▸(*b*) and 2 ▸(*c*)]. The binding cavity of esterases typically exhibits a negatively charged electrostatic potential distribution, serving as an electrostatic catapult (Neves Petersen *et al.*, 2001 ▸). In contrast, the binding cavity of PsEst3 exhibited a positively charged electrostatic potential distribution [Fig. 2 ▸(*d*)]. In addition, the degree of conservation of the residues was represented as a surface model via the multiple sequence alignment of 250 species that were selected based on the *BLAST* results for PsEst3 in the UniProt database [Fig. 2 ▸(*e*)]. The residues located in the active site, including the catalytic triad (Ser128, Asp199 and His227), were strictly conserved. In addition, Arg44 was observed to be highly conserved, representing another unique characteristic of PsEst3.

### 3.4. Sequence and structural similarity analyses

**Figure 3:**
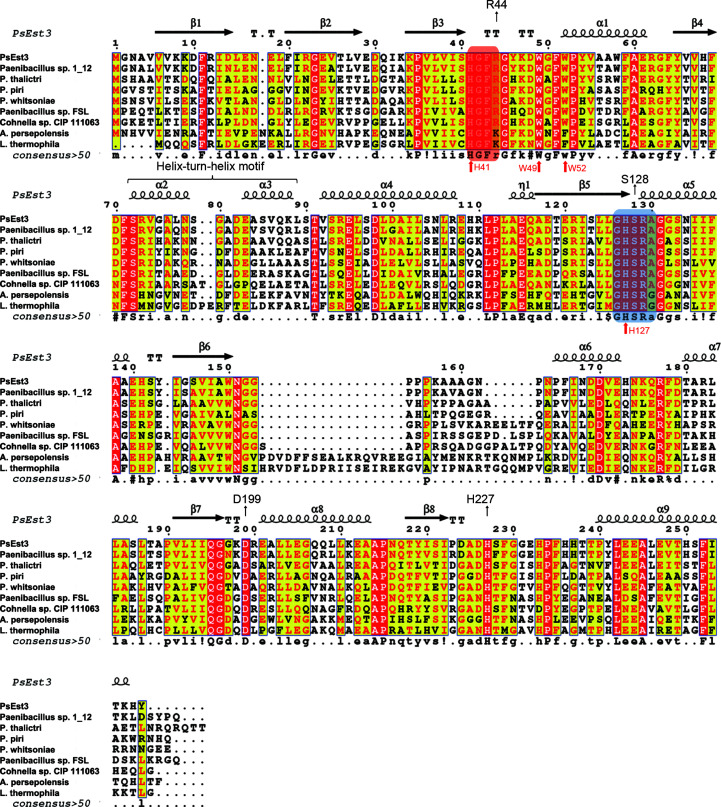
Multiple sequence alignment of PsEst3 with eight homologs [*Paenibacillus* sp. 1_12 (UniProt accession No. A0A1I4E7Z0), *P. thalictri* (A0A4Q9DLG8), *P. piri* (A0A4R5KT22), *P. whitsoniae* (A0A430JH40), *Paenibacillus* sp. FSL H7-0357 (A0A089I8J6), *Cohnella* sp. CIP 111063 (A0A231R2L3), *Alteribacillus persepolensis* (A0A1G8AGB0) and *Lihuaxuella thermophila* (A0A1H8C781)] obtained from the *BLAST* results of PsEst3 using the UniProt database. The last two homologs (A0A1G8E3W4 and A0A1H8C781) that contain a GHSRG motif instead of GHSRA were added to the alignment. The secondary structure of PsEst3 is shown above the sequence. The pentapeptide sequence (GHSRA/G) and oxyanion hole-forming sequence (HGFR/K) are highlighted with blue and red rectangles, respectively. The catalytic triads and residues forming the acyl-binding pocket are indicated with black and red arrows, respectively.

**Figure 4:**
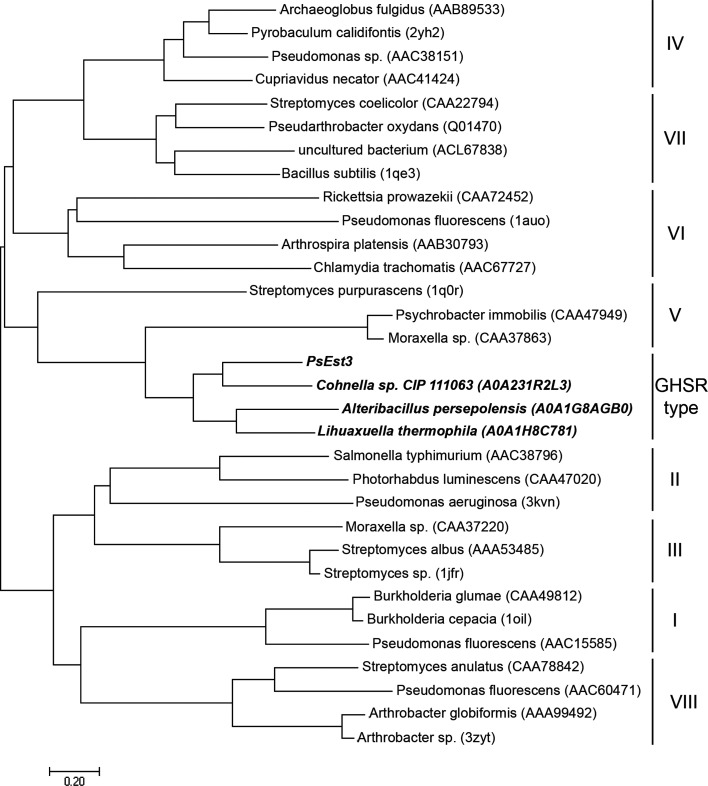
Phylogenetic tree of PsEst3 with lipases/esterases of other classes. The evolutionary history was inferred using the neighbor-joining method based on the amino-acid sequence of PsEst3. The lipase/esterase enzymes previously classified into classes I–VIII were used as input together with PsEst3 and three other homologs (UniProt accession Nos. A0A231R2L3, A0A1G8E3W4 and A0A1H8C781). Evolutionary analyses were conducted in *MEGA*7. The tree is drawn to scale, with branch lengths in the same units as those of the evolutionary distances used to infer the phylogenetic tree. GenBank, UniProt or PDB accession codes of the reference sequences are shown in parentheses.

Ser128 was positioned in the pentapeptide sequence 126-GHSRA/G-130 and the oxyanion hole was composed of 41-HGFR/K-44 (Fig. 3 ▸). Although a previous study reported that the 35 families of esterases each have their own unique pentapeptide sequence containing the catalytic serine (Hitch & Clavel, 2019 ▸), the pentapeptide sequence of PsEst3, 126-GHSRA-130, was not found in any of the defined families (Fig. 3 ▸). The PsEst3 phylogenetic tree was generated to assess its relationships to eight families (Fig. 4 ▸; Arpigny & Jaeger, 1999 ▸). PsEst3 was related to the class V family but was differentiated in the early stages, showing r.m.s.d.s of 2.1 and 2.3 Å when its structure was compared with those of aclacinomycin methylesterase (RdmC) from *Streptomyces purpurascens* (PDB entry 1q0r; Jansson *et al.*, 2003 ▸) and haloalkane dehalogenase from *Xanthobacter autotrophicus* (XaHDH; PDB entry 2dhd; Verschueren *et al.*, 1993 ▸), respectively [Supplementary Figs. S2(*a*)–S2(*d*)]. The large α-helix bundle domain of RdmC and XaHDH, which is an alternative form of a lid for interfacial activation, was detected in the simplified form of α6 in PsEst3. The superposition of PsEst3 onto class VI carboxylesterase from *Pseudomonas fluorescens* (PfEst) results in an r.m.s.d. of 1.9 Å [Supplementary Figs. S2(*a*)–S2(*d*)] (Kim *et al.*, 1997 ▸). An expanded motif including two β-sheets was observed in the cognate region of PfEst, which was replaced with the HtH motif in PsEst3. Thus, the phylogenetic tree of PsEst3 and structural comparisons showed that PsEst3, class V lipases and VI carboxyl esterases shared a common ancestor a long time ago but have evolved separately.

Feruloyl esterase from *Butyrivibrio proteoclasticus* (BpEst1E; Goldstone *et al.*, 2010 ▸) and cinnamoyl esterase from *Lactobacillus johnsonii* (LjEst; Lai *et al.*, 2011 ▸) were identified by a *DALI* server search (Holm, 2020 ▸). Although dinelactone hydrolase from *Trichormus variabilis* (TvDEH; PDB entry 2o2g) was also identified as similar to PsEst3, the mechanism by which it hydrolyzes dienelactone has not been established. Hence, in this study, the active-site residues of dienelactone hydrolase from *Pseudomonas knackmussii* (PkDLH; PDB entry 1din), in addition to TvDEH, were structurally compared with those of PsEst3 (Cheah *et al.*, 1993 ▸; Pathak & Ollis, 1990 ▸). The overall folds of the three enzymes were similar to that of PsEst3; however, certain peripheral differences were observed (Supplementary Fig. S3) and the pentapeptide sequence of each enzyme was not fully aligned with that of PsEst3 in the multiple sequence alignment [Supplementary Fig. S4(*a*)]. Moreover, within the structures of BpEst1E and LjEst α6 was replaced by two α-helices and four β-strands in the counterparts, forming a partial lid domain [Supplementary Figs. S3(*a*) and S3(*b*)]. The HtH motif was also substituted by a motif comprising an α-helix and a short loop. The complexity of the lid domain affects the overall shape of the binding pocket and its channel. The binding pocket of PsEst3 is connected to three different channels formed by α6 and the main body. However, the partial lid domain of BpEst1E and LjEst lacks the channel located in the deep region of the binding pocket. In contrast, a simplification of the region was observed in TvDEH [Supplementary Fig. S3(*d*)] with the region not being covered by the short α-helix, thus leaving the active site completely exposed to the solvent.

In terms of substrate specificity, we designed a competition assay using putative substrate analogs. We observed a change in enzyme activity caused by competitive inhibition by the putative substrate analogs. In the structure homology search using the *DALI* server, the enzymes with the best-matched structures to that of PsEst3 were feruloyl esterase and cinnamoyl esterase. However, the results of the enzyme-activity assay using *p*NP-C2 revealed that PsEst3 was not inhibited by phenolic compounds such as ferulic acid, vanillic acid or cinnamic acid, which were expected to serve as substrates or products [Supplementary Fig. S4(*b*)]. This result suggests that this enzyme might be not related to feruloyl esterase or cinnamoyl esterase (Crepin *et al.*, 2003 ▸).

### 3.5. Binding mode of ligands in the crystal structure

**Figure 5:**
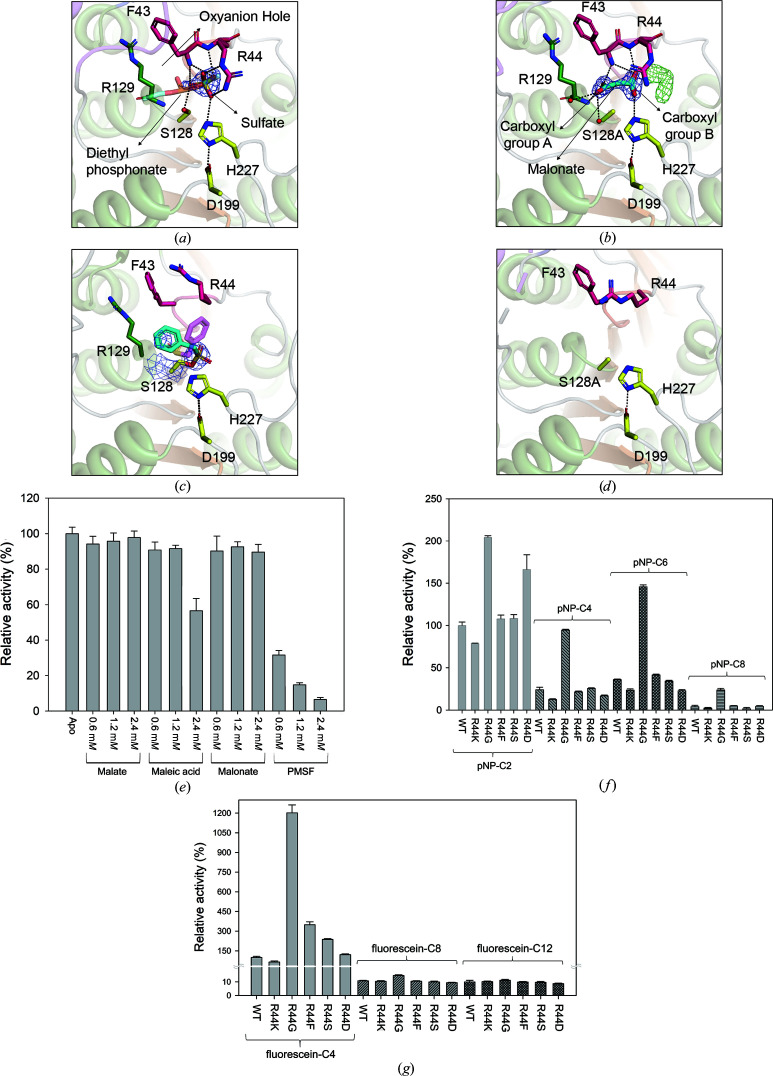
Binding modes of various ligands. The active sites of the sulfate-bound structure (*a*), malonate (MLA)-bound S128A mutant structure (*b*), PMSF-bound structure (*c*) and S128A mutant structure (*d*) are shown. The overall color scheme of PsEst3 is the same as that in Fig. 1 ▸(*a*). The 2*F*_o_ − *F*_c_ map of sulfate and malonate (blue) and the *F*_o_ − *F*_c_ map of unidentified ligands (green) are contoured at the 2σ level except for in the PMSF complex (1.5σ level). The hydrogen bonds between ligands and PsEst3 are represented as black dotted lines. The transition-state analog (diethyl phosphonate)-bound structure of an extracellular triglyceride lipase (PDB entry 4tgl) and the PMSF-bound structure of EstE5 (PDB entry 3h17) are superposed on PsEst3, and the diethyl phosphonate in PDB entry 4tgl and the PMSF in PDB entry 3h17 are represented as transparent stick models in (*a*) and (*c*), respectively. (*e*) Inhibition activity by malate (salt form), maleic acid, malonate and PMSF. The respective ligand was added in the concentration range 0.6–2.4 m*M*. (*f*) The *p*NP-ester (C2, C4, C6 and C8) hydrolysis activity measured with various Arg44 variants (R44K, R44G, R44F, R44S and R44D). (*g*) Relative activities of fluorescein derivatives (C4, C8 and C12) measured with various Arg44 variants.

Two unintentionally complexed ligands that were derived from the crystallization solution were detected in the PsEst3 structures: a sulfate and a malonate. Structural superposition of the sulfate-bound structure and the malonate-bound structure of the S128A mutant with the apo structure of the S128A mutant revealed r.m.s.d.s of 0.4 and 0.3 Å, respectively, indicating that the overall conformational changes caused by the ligands were not critical. In the sulfate-bound structure, a sulfate molecule occupied the active site near Ser128, interacting with Phe43, Arg44, Ser128 and His227 [Figs. 5 ▸(*a*) and 5 ▸(*b*)]. Compared with diethyl phosphonate (Derewenda *et al.*, 1992 ▸), which mimicked the transition state interacting with the oxyanion hole, sulfate exhibited an approximate shift of 2.6 Å caused by hydrogen bonds between an O atom of sulfate and the N atoms of the 43-FR-44 main chain. Another O atom of sulfate was located near the oxyanion hole comprising the backbone N atoms of Arg129 and Phe43; however, its orientation did not directly mimic that of diethyl phosphonate. Two carboxyl groups of the malonate molecule exhibited distinct binding modes similar to those of sulfate and dimethyl phosphonate simultaneously. One O atom of carboxyl group A was located in the oxyanion hole, forming hydrogen bonds to Phe43 and Arg129, whereas another O atom interacted with two water molecules, similar to the alcohol moiety of the substrate [Fig. 5 ▸(*b*) and Supplementary Fig. S5(*b*)]. Carboxyl group B exhibited a binding mode similar to that of sulfate. One O atom of carboxyl group B also formed hydrogen bonds to the N atoms of the 43-FR-44 main chain, and the other O atom interacted with His227. The binding mode of PMSF in the PsEst3 structure differed from that in the PMSF-complexed EstE5 structure (PDB entry 3h17; Nam *et al.*, 2009 ▸) [Fig. 5 ▸(*c*) and Supplementary Fig. S5(*c*)]. The benzene ring of PMSF in the PsEst3 structure protruded into the alcohol-binding pocket, whereas that in EstE5 was exposed to the solvent region. To confirm whether malonate is a true substrate of PsEst3, the inhibitory effects of binding ligands (malate, maleic acid and malonate) on the enzyme activity were investigated using the substrate *p*NP-C2. Malate and malonate did not exhibit meaningful inhibition of *p*NP esters, but 2.4 m*M* maleic acid caused an approximately 40% decrease in activity. This may be due to the carbon bridge of maleic acid containing one more C atom than that of malonate, creating a region with extra density near carboxyl group B, which resembles an acetate molecule [Fig. 5 ▸(*b*)]. PMSF also inhibited over 60% of the activity at a concentration of 0.6 m*M* [Fig. 5 ▸(*e*)].

The Arg44 residue formed hydrogen bonds and hydrophobic interactions with the ligands and physically separated the ligand cavity from the solvent region in the sulfate- and malonate-bound structures [Figs. 5 ▸(*a*) and 5 ▸(*b*)]. However, Arg44 in the apo form of the S128A mutant and PMSF-bound structures was completely disordered, exposing the cavity to the solvent [Fig. 5 ▸(*d*)]. Thus, to investigate the role of Arg44 in the activity of PsEst3, R44K, R44G, R44F, R44S and R44D variants were tested with *p*NP esters or fluorescein derivatives of different lengths [Figs. 5 ▸(*f*) and 5 ▸(*h*)]. In the assay involving *p*NP-C2, all variants, excluding R44G and R44D, exhibited superior activity to wild-type PsEst3. In particular, R44G exhibited a twofold higher activity than wild-type PsEst3. This could be due to a solvent-exposed cavity resulting from the elimination of the bulky side chain of Arg44. In the case of *p*NP-C4, *p*NP-C6 and *p*NP-C8 the activity of R44G was significantly increased compared with that of wild-type PsEst3 and other variants. Additionally, we measured the hydrolase activity of PsEst3 against fluorescein derivatives (C4, C8 and C12) to assess the effect of a bulky alcoholic moiety on the substrate. The activity of R44G increased 12-fold, indicating that the effect of R44G was amplified when hydrolyzing a substrate with a large alcoholic moiety. These findings indicate that Arg44 seals the cavity from the solvents and controls the binding of substrates.

### 3.6. PsEst3 activity assays

**Figure 6:**
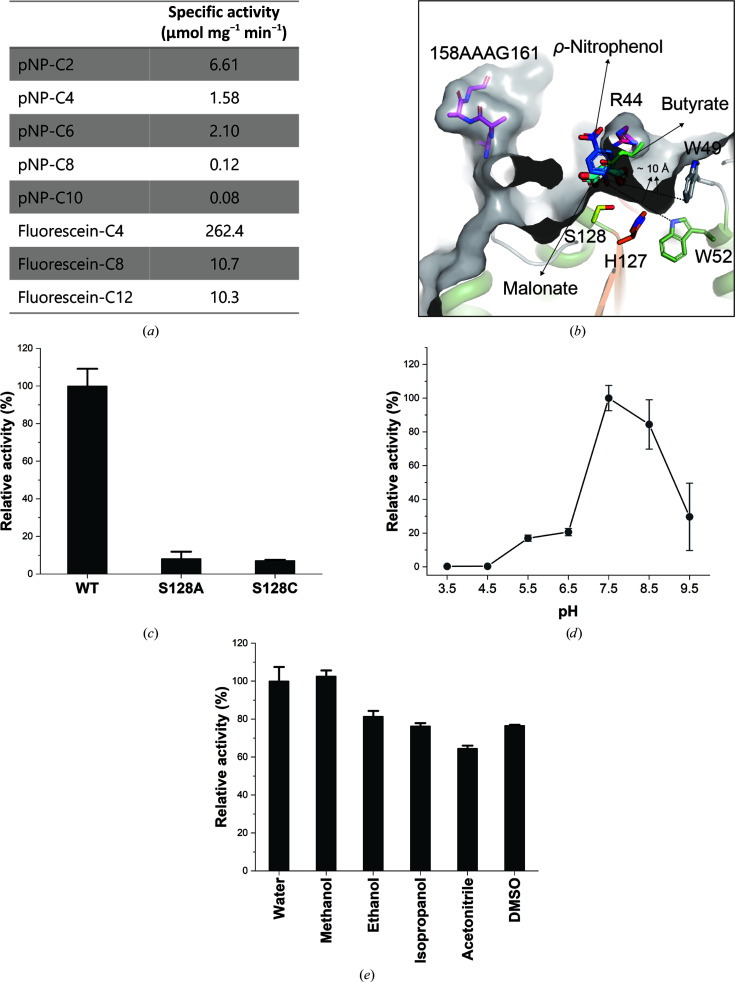
(*a*) Specific activity of PsEst3 against *p*-nitrophenol (*p*NP) esters and fluorescein derivatives. (*b*) Comparison of the ligand-binding mode. The *p*-­nitrophenol-bound structure of Est22 (PDB entry 5hc2) and the butyrate-bound structure of thermophilic esterase (PDB entry 4uhf) are superposed on PsEst3 to compare ligand orientations (Huang *et al.*, 2016 ▸; Sayer *et al.*, 2015 ▸). *p*-Nitrophenol and butyrate are represented as blue and light green sticks, respectively. Malonate in PsEst3 is shown as relatively thick cyan sticks. The residues that form the binding pocket for an acid are also shown as stick models. The distances between the carbon center of carboxyl group A of malonate and Trp49 and Trp52 are shown as black dotted lines (10.0 and 10.1 Å, respectively). (*c*) *p*NP-C2 hydrolysis activity of PsEst3 Ser128 variants. The effects of varying the pH value (*d*) and organic solvents (*e*) on PsEst3 activity are shown. *p*NP-C2 was used as the substrate.

The specific activities of *p*NP esters (*p*NP-C2, *p*NP-C4, *p*NP-C6, *p*NP-C8 and *p*NP-C10) and fluorescein diesters (fluorescein-diC4, fluorescein-diC8 and fluorescein-diC12) were measured to determine the preferred chain lengths. The activity of PsEst3 during the hydrolysis of *p*NP esters decreased with increasing *p*NP ester chain length, with the exception of *p*NP-C6 [Fig. 6 ▸(*a*)]. The distance of the acyl-binding pocket of PsEst3 (between the carbon center of carboxyl group A and Trp49 and Trp52) was approximately 10 Å [Fig. 6 ▸(*b*)], which is equivalent to approximately twice the length of butyrate (3.8 Å; Sayer *et al.*, 2015 ▸). This was consistent with the fact that *p*NP-C8 and *p*NP-C10 exhibited only 1–2% of the hydrolytic activity of *p*NP-C2 due to steric hindrance by Trp49 and Trp52. These residues were highly conserved among the species selected from PsEst3 *BLAST* results (Fig. 3 ▸). Therefore, PsEst3 was shown to be specialized for the hydrolysis of C2–C6 esters.

PsEst3 also showed a significant similarity to dienelactone hydrolase and other α/β hydrolases of known structure, as shown by the *DALI* search results. Typically, dienelactione hydrolase contains a catalytic triad containing a cysteine residue, instead of a serine, as the nucleophile (Lenfant *et al.*, 2013 ▸). The S128A and S128C variants were prepared to investigate whether PsEst3 exhibits the same mechanism as that of dienelactone hydrolase or a classical esterase. Mutational analysis revealed that the activities of both variants were <10% that of the wild type [Fig. 6 ▸(*c*)]. However, the mechanism of TvDEH is not yet fully understood, even though the structural similarity between PsEst3 and TvDEH shows an r.m.s.d. of 1.85 Å. We structurally analyzed the active-site residues of PkDLH in addition to TvDEH; a detailed hypothesis on the reaction mechanism of PkDLH has previously been reported (Cheah *et al.*, 1993 ▸). Three residues (Arg81, Arg206 and Ser228) in the structure of PkDLH are involved in substrate binding. Among them, Arg81 and Arg206 of PkDLH, which are located in the distal region of the active site, play an important role in stabilizing the carboxylate moiety of dienelactam through ionic interaction. In the superimposed structure [Supplementary Fig. S3(*e*)], both Arg51 of TvDEH and Arg206 of PkDLH are in close proximity. The distance between the C^α^ atoms of the two residues is approximately ∼4.5 Å. However, PsEst3 does not have residues corresponding to Arg81 and Arg206 of PkDLH. The nearest positively charged residue of PsEst3 corresponding to Arg81 of PkDLH is Arg44 in the oxyanion hole sequence, while the relative distance between C^α^ atoms of the two residues is approximately ∼6.3 Å. The Ser203 residue of PkDLH interacts with the carboxylate of dienelactam via a hydrogen bond, which corresponds to Ser228 of PsEst3. The distance between C^α^ atoms of the two residues is approximately 1.7 Å. Based on the substrate-binding mode of PkDLH, our structural analysis suggested that the active-site conformation of PsEst3 is different from the active-site cleft of PkDLH and TvDEH involved in identifying and stabilizing the function of the substrate dienelactone.

To investigate the effect of pH and chemicals on enzyme activity, the activity was assessed at various pH values and in the presence of organic solvents [Figs. 6 ▸(*d*) and 6 ▸(*e*)]. The optimal pH for PsEst3 was 7.5, similar to those of other esterases. Evaluation of the effects of different organic solvents on enzyme activity provides important insights regarding the factors that may affect catalytic function, such as conformational modifications of the enzyme structure and substrate solubility, in industrial usage. However, in this study the activity of PsEst3 was not significantly affected by different organic solvents.

## 4. Conclusion

The esterase PsEst3 was investigated to understand its structural and biochemical characteristics (Kim *et al.*, 2018 ▸; Shin *et al.*, 2020 ▸). The structure of PsEst3 was determined at 1.8 Å resolution and was found to share certain structural features with class V and VI esterases (Supplementary Fig. S2). However, the phylogenetic tree constructed based on the sequence homology of lipases/esterases showed that PsEst3 diverged from an early common ancestor (Fig. 4 ▸). Furthermore, *BLAST* using the UniProt database identified proteins with a pentapeptide sequence (GHSRA) identical to that of PsEst3, which is annotated as a dienelactone hydrolase. Sequence analysis further identified the catalytic serine residue within the pentapeptide 126-GHSRA-130 as the conserved motif G*x*S*x*G. Nevertheless, the unique characteristic of the 126-GHSRA-130 pentapeptide sequence, the oxyanion hole-forming sequence 41-HGFR-44, the positively charged electrostatic potential of the active site, which may function as a landing strip for negatively charged ligands, and the nonconserved dienelactone recognition residues in PsEst3 provide sufficient evidence to classify PsEst3 into a new family of esterases.

The Arg44 residue is another feature of PsEst3 that distinguishes it from other esterases and functions to partially cover the active site, thereby inhibiting the hydrolytic function of PsEst3. However, the specific role of Arg44 in the activity of PsEst3 remains unclear. Nevertheless, we concluded that it is customized for a substrate that has not yet been identified. Moreover, the results of the substrate-specificity assay indicate that the specific hydrolysis activity of fluorescein diesters was 40-fold higher than that of *p*NP esters, suggesting that the true PsEst3 substrates might contain bulky alcoholic moieties [Fig. 6 ▸(*a*)]. Together, in terms of protein engineering, the increased activity of R44G and the moderate activity of PsEst3 at low temperatures enhance the value of PsEst3 as an industrial biocatalyst.

## Supplementary Material

PDB reference: PsEst3, wild type, 7v8u

PDB reference: complex with phenylmethylsulfonyl fluoride, 7v8x

PDB reference: S128A mutant, 7v8v

PDB reference: S128A mutant, complex with malonate, 7v8w

Supplementary Figures. DOI: 10.1107/S2052252523001562/jt5064sup1.pdf

## Figures and Tables

**Table 1:** Data-collection and refinement statistics Values in parentheses are for the highest resolution shell.

	Wild type with sulfate	S128A mutant with malonate	Wild type with PMSF	Apo S128A mutant
PDB code	7v8u	7v8w	7v8x	7v8v
Data collection				
Wavelength (Å)	0.9796	0.97942	0.97933	0.9795
Space group	*P*4_1_32	*P*4_1_32	*P*4_1_32	*P*4_1_32
*a*, *b*, *c* (Å)	145.13, 145.13, 145.13	144.24, 144.24, 144.24	144.77, 144.77, 144.77	143.61, 143.61, 143.61
α, β, γ (°)	90.00, 90.00, 90.00	90.00, 90.00, 90.00	90.00, 90.00, 90.00	90.00, 90.00, 90.00
Resolution (Å)	50.00–2.25 (2.38–2.25)	50.00–1.80 (1.83–1.80)	50.00–2.34 (2.38–2.34)	50.00–2.40 (2.54–2.40)
*R*_merge_ †	0.081 (0.204)	0.061 (0.544)	0.075 (0.524)	0.079 (0.694)
CC_1/2_	0.999 (0.985)	1.000 (0.509)	1.000 (0.369)	0.999 (0.873)
〈*I*/σ(*I*)〉	26.1 (9.04)	44.5 (2.67)	60.6 (4.57)	21.2 (3.00)
Completeness (%)	99.9 (99.9)	100 (100)	99.8 (99.8)	99.7 (99.4)
Multiplicity	16.7 (7.68)	33.5 (22.1)	30.4 (14.0)	7.4 (7.5)
Refinement				
Resolution (Å)	48.38–2.25	45.6–1.80	35.1–2.34	34.8–2.40
No. of reflections	25484	48026	22402	20442
*R*_work_/*R*_free_	0.179/0.210	0.188/0.208	0.225/0.257	0.194/0.226
No. of atoms				
Protein	2040	2010	2001	1997
Ligand/ion	14	19	10	0
Solvent	187	142	55	139
Ramachandran plot (%)				
Favored regions	98.4	96.4	94.88	96.8
Allowed regions	1.6	3.6	5.1	3.2
Outlier regions	0	0	0	0
*B* factors (Å^2^)				
Protein	30.7	34.9	54.5	44.7
Ligand/ion	42.8	42.6	74.0	—
Solvent	38.6	40.5	51.8	50.5
R.m.s. deviations				
Bond lengths (Å)	0.010	0.009	0.008	0.009
Bond angles (°)	1.069	1.000	1.123	1.002

† *R*_merge_ = 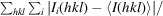 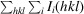 .

## References

[bb1] Afonine, P. V., Grosse-Kunstleve, R. W., Echols, N., Headd, J. J., Moriarty, N. W., Mustyakimov, M., Terwilliger, T. C., Urzhumtsev, A., Zwart, P. H. & Adams, P. D. (2012). *Acta Cryst.* D**68**, 352–367. 10.1107/S0907444912001308 PMC3322595 22505256

[bb2] Arpigny, J. L. & Jaeger, K. E. (1999). *Biochem. J.* **343**, 177–183. PMC1220539 10493927

[bb3] Bartsch, S., Kourist, R. & Bornscheuer, U. T. (2008). *Angew. Chem. Int. Ed.* **47**, 1508–1511. 10.1002/anie.200704606 18203225

[bb4] Bordes, F., Barbe, S., Escalier, P., Mourey, L., André, I., Marty, A. & Tranier, S. (2010). *Biophys. J.* **99**, 2225–2234. 10.1016/j.bpj.2010.07.040 PMC3042558 20923657

[bb5] Bornscheuer, U. T. (2002). *FEMS Microbiol. Rev.* **26**, 73–81. 10.1111/j.1574-6976.2002.tb00599.x 12007643

[bb6] Cheah, E., Ashley, G. W., Gary, J. & Ollis, D. (1993). *Proteins*, **16**, 64–78. 10.1002/prot.340160108 8497485

[bb7] Crepin, V. F., Faulds, C. B. & Connerton, I. F. (2003). *Biochem. J.* **370**, 417–427. 10.1042/BJ20020917 PMC1223187 12435269

[bb8] Derewenda, U., Brzozowski, A. M., Lawson, D. M. & Derewenda, Z. S. (1992). *Biochemistry*, **31**, 1532–1541. 10.1021/bi00120a034 1737010

[bb9] Emsley, P., Lohkamp, B., Scott, W. G. & Cowtan, K. (2010). *Acta Cryst.* D**66**, 486–501. 10.1107/S0907444910007493 PMC2852313 20383002

[bb10] Gerday, C., Aittaleb, M., Bentahir, M., Chessa, J.-P., Claverie, P., Collins, T., D’Amico, S., Dumont, J., Garsoux, G., Georlette, D., Hoyoux, A., Lonhienne, T., Meuwis, M.-A. & Feller, G. (2000). *Trends Biotechnol.* **18**, 103–107. 10.1016/s0167-7799(99)01413-4 10675897

[bb11] Goldstone, D. C., Villas-Bôas, S. G., Till, M., Kelly, W. J., Attwood, G. T. & Arcus, V. L. (2010). *Proteins*, **78**, 1457–1469. 10.1002/prot.22662 20058325

[bb12] Grochulski, P., Li, Y., Schrag, J. D., Bouthillier, F., Smith, P., Harrison, D., Rubin, B. & Cygler, M. (1993). *J. Biol. Chem.* **268**, 12843–12847. 8509417

[bb13] Guglielmetti, S., De Noni, I., Caracciolo, F., Molinari, F., Parini, C. & Mora, D. (2008). *Appl. Environ. Microbiol.* **74**, 1284–1288. 10.1128/AEM.02093-07 PMC2258599 18165367

[bb14] Hitch, T. C. A. & Clavel, T. (2019). *PeerJ*, **7**, e7249. 10.7717/peerj.7249 PMC6622161 31328034

[bb15] Holm, L. (2020). *Protein Sci.* **29**, 128–140. 10.1002/pro.3749 PMC6933842 31606894

[bb16] Houde, A., Kademi, A. & Leblanc, D. (2004). *Appl. Biochem. Biotechnol.* **118**, 155–170. 10.1385/abab:118:1-3:155 15304746

[bb17] Huang, J., Huo, Y.-Y., Ji, R., Kuang, S., Ji, C., Xu, X.-W. & Li, J. (2016). *Sci. Rep.* **6**, 28550. 10.1038/srep28550 PMC4916508 27328716

[bb18] Jansson, A., Niemi, J., Mäntsälä, P. & Schneider, G. (2003). *J. Biol. Chem.* **278**, 39006–39013. 10.1074/jbc.M304008200 12878604

[bb19] Kim, H., Park, A. K., Lee, J. H., Shin, S. C., Park, H. & Kim, H.-W. (2018). *Acta Cryst.* F**74**, 367–372. 10.1107/S2053230X18007525 PMC5987746 29870022

[bb20] Kim, H.-K., Liu, J.-W., Carr, P. D. & Ollis, D. L. (2005). *Acta Cryst.* D**61**, 920–931. 10.1107/S0907444905009042 15983415

[bb21] Kim, H.-W., Wi, A. R., Jeon, B. W., Lee, J. H., Shin, S. C., Park, H. & Jeon, S.-J. (2015). *Biotechnol. Lett.* **37**, 1887–1893. 10.1007/s10529-015-1860-y 26003095

[bb22] Kim, K. K., Song, H. K., Shin, D. H., Hwang, K. Y., Choe, S., Yoo, O. J. & Suh, S. W. (1997). *Structure*, **5**, 1571–1584. 10.1016/s0969-2126(97)00306-7 9438866

[bb23] Kumar, S., Stecher, G., Li, M., Knyaz, C. & Tamura, K. (2018). *Mol. Biol. Evol.* **35**, 1547–1549. 10.1093/molbev/msy096 PMC5967553 29722887

[bb24] Lai, K. K., Stogios, P. J., Vu, C., Xu, X., Cui, H., Molloy, S., Savchenko, A., Yakunin, A. & Gonzalez, C. F. (2011). *PLoS One*, **6**, e23269. 10.1371/journal.pone.0023269 PMC3158066 21876742

[bb25] Laizure, S. C., Herring, V., Hu, Z., Witbrodt, K. & Parker, R. B. (2013). *Pharmacotherapy*, **33**, 210–222. 10.1002/phar.1194 PMC4572478 23386599

[bb26] Lenfant, N., Hotelier, T., Velluet, E., Bourne, Y., Marchot, P. & Chatonnet, A. (2013). *Nucleic Acids Res.* **41**, D423–D429. 10.1093/nar/gks1154 PMC3531081 23193256

[bb27] Li, C., Hassler, M. & Bugg, T. D. (2008). *ChemBioChem*, **9**, 71–76. 10.1002/cbic.200700428 18058773

[bb28] Lian, J., Nelson, R. & Lehner, R. (2018). *Protein Cell*, **9**, 178–195. 10.1007/s13238-017-0437-z PMC5818367 28677105

[bb29] Liu, H. & Naismith, J. H. (2008). *BMC Biotechnol.* **8**, 91. 10.1186/1472-6750-8-91 PMC2629768 19055817

[bb30] Nam, K. H., Kim, S.-J., Priyadarshi, A., Kim, H. S. & Hwang, K. Y. (2009). *Biochem. Biophys. Res. Commun.* **389**, 247–250. 10.1016/j.bbrc.2009.08.123 19715665

[bb31] Neves Petersen, M. T., Fojan, P. & Petersen, S. B. (2001). *J. Biotechnol.* **85**, 115–147. 10.1016/s0168-1656(00)00360-6 11165360

[bb32] Ollis, D. L., Cheah, E., Cygler, M., Dijkstra, B., Frolow, F., Franken, S. M., Harel, M., Remington, S. J., Silman, I., Schrag, J., Sussman, J. L., Verschueren, K. H. G. & Goldman, A. (1992). *Protein Eng. Des. Sel.* **5**, 197–211. 10.1093/protein/5.3.197 1409539

[bb33] Otwinowski, Z. & Minor, W. (1997). *Methods Enzymol.* **276**, 307–326. 10.1016/S0076-6879(97)76066-X 27754618

[bb34] Panda, T. & Gowrishankar, B. S. (2005). *Appl. Microbiol. Biotechnol.* **67**, 160–169. 10.1007/s00253-004-1840-y 15630579

[bb35] Pathak, D. & Ollis, D. (1990). *J. Mol. Biol.* **214**, 497–525. 10.1016/0022-2836(90)90196-s 2380986

[bb36] Robert, X. & Gouet, P. (2014). *Nucleic Acids Res.* **42**, W320–W324. 10.1093/nar/gku316 PMC4086106 24753421

[bb37] Saitou, N. & Nei, M. (1987). *Mol. Biol. Evol.* **4**, 406–425. 10.1093/oxfordjournals.molbev.a040454 3447015

[bb38] Sayer, C., Isupov, M. N., Bonch-Osmolovskaya, E. & Littlechild, J. A. (2015). *FEBS J.* **282**, 2846–2857. 10.1111/febs.13326 26011036

[bb39] Schrag, J. D., Li, Y. G., Wu, S. & Cygler, M. (1991). *Nature*, **351**, 761–764. 10.1038/351761a0 2062369

[bb40] Shin, S. C., Choi, W., Lee, J., Kim, H. J. & Kim, H.-W. (2020). *3 Biotech*, **10**, 480. 10.1007/s13205-020-02474-0 PMC7573059 33094089

[bb41] Sztajer, H., Lünsdorf, H., Erdmann, H., Menge, U. & Schmid, R. (1992). *Biochim. Biophys. Acta*, **1124**, 253–261. 10.1016/0005-2760(92)90137-k 1576166

[bb42] Verschueren, K. H. G., Seljée, F., Rozeboom, H. J., Kalk, K. H. & Dijkstra, B. W. (1993). *Nature*, **363**, 693–698. 10.1038/363693a0 8515812

