# Supplementary material for: Structural and biochemical insights into PsEst3, a new GHSR-type esterase obtained from Paenibacillus sp. R4
Source: IUCrJ. 2023 Feb 28;10(Pt 2):220–32. doi: 10.1107/S2052252523001562 (PMC9980389; doi:10.1107/S2052252523001562)
Supplement: Supplementary file 1 [file m-10-00220-sup1.pdf]

IUCrJ

Volume 10 (2023)

Supporting information for article:

Structural and biochemical insights into PsEst3, a new GHSR-type esterase obtained from *Paenibacillus* sp. R4

Jonghyeon Son, Woong Choi, Hyun Kim, Minseo Kim, Jun Hyuck Lee, Seung Chul Shin and Han-Woo Kim

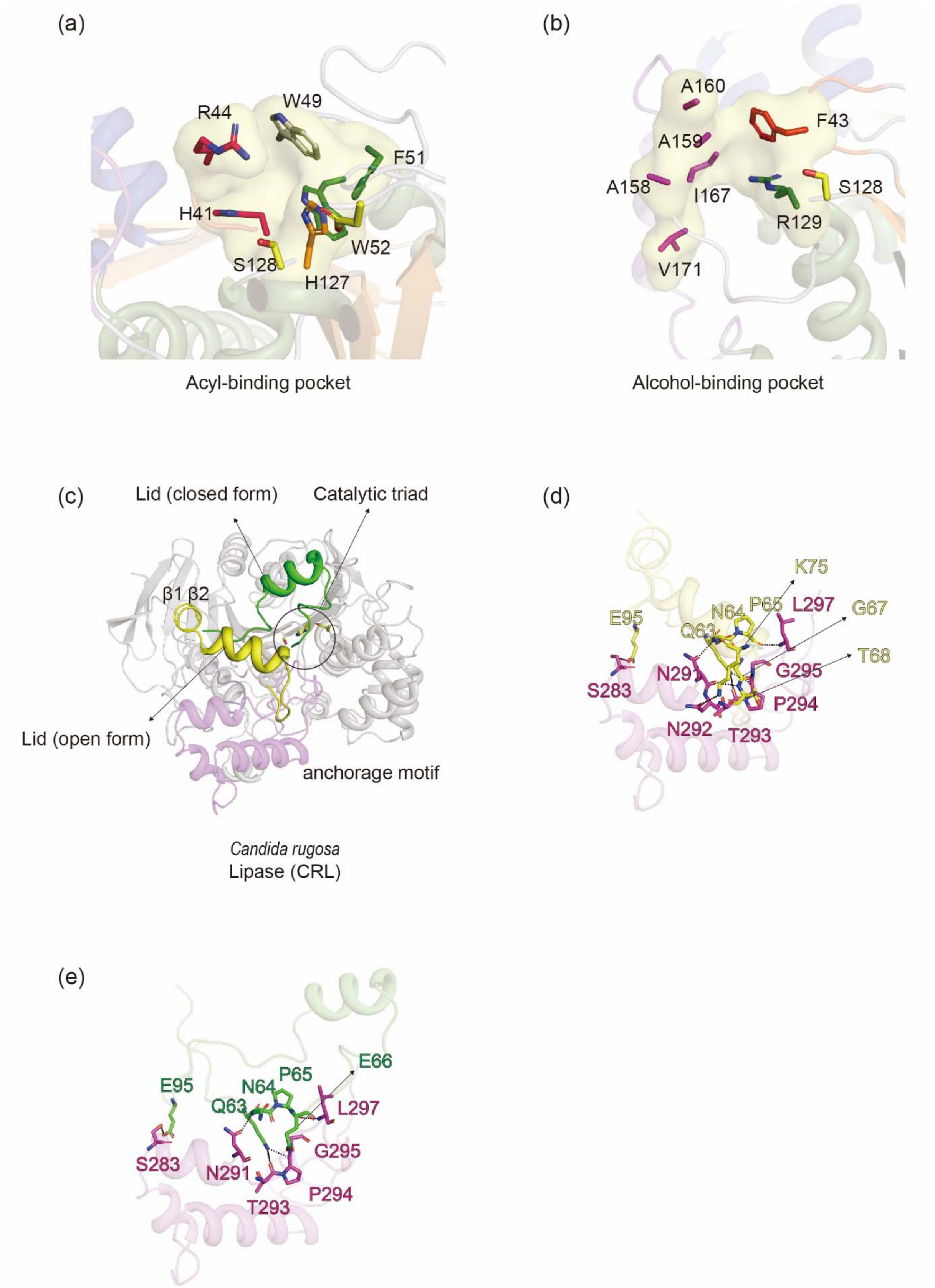

Figure S1 Residues of ligand-binding pockets and the lid domain of *Candida rugosa* ligase (CRL). An acyl-binding pocket (a) and alcohol-binding pocket (b) of the sulfate-bound structure. The residues

that contribute to pocket formation are represented as stick and surface models. S128 and H127 are represented as a *yellow* stick model. (c) Crystal structure of CRL (1crl) is represented as a cartoon model. The lid domain and anchorage motif of CRL appear *yellow* and *magenta*, respectively. The closed state structure of CRL (1trh) is superposed on the open state of CRL; the lid domain appears *green*. Catalytic triads are represented as *yellow* sticks. (d and e) Magnified view of the lid domain and anchorage motif of the open (d) and closed (e) states. The residues that take part in the inter-motif interaction between the lid domain and anchorage motif are represented as a stick model.

(a)

Class	PDB entry	R.m.s.d.	Residues
I	1oil	2.37	173
II	3kvn	3.53	142
III	1jfr	1.74	190
IV	2yh2	2.40	219
V	1q0r	2.12	194
	2dhd	2.28	202
VI	1auo	1.91	169
VII	1qe3	2.45	219
VIII	3zyt	4.18	103

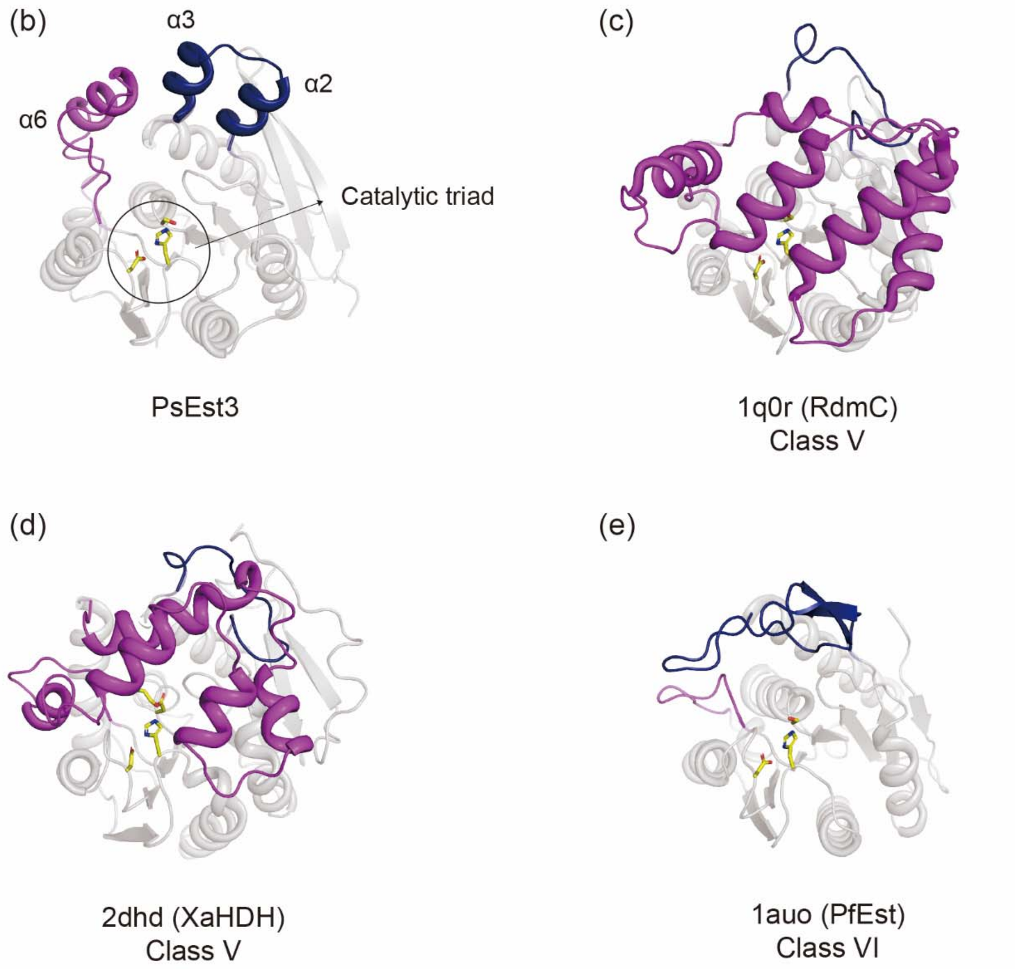

Figure S2 Comparison of PsEst3 with esterase various classes. (a) R.m.s.d. values for the superposition of PsEst3 into esterases of classes I–VIII and the number of residues used to calculate r.m.s.d. values are listed. (b)–(e) Class V lipases [(c); aclacinomycin methylesterase (RdmC, 1q0r) from *Streptomyces purpurascens*, and (d); haloalkane dehalogenase from *Xanthobacter autotrophicus* (XaHDH, 2dhd)] and class VI esterase [(e); carboxylesterase from *Pseudomonas fluorescens* (PfEst, 1auo)] are superposed to PsEst3 (b) and represented as cartoon models. α6 and the HtH motif of PsEst3 and the corresponding regions of other esterases are highlighted as *magenta* and *dark blue*, respectively.

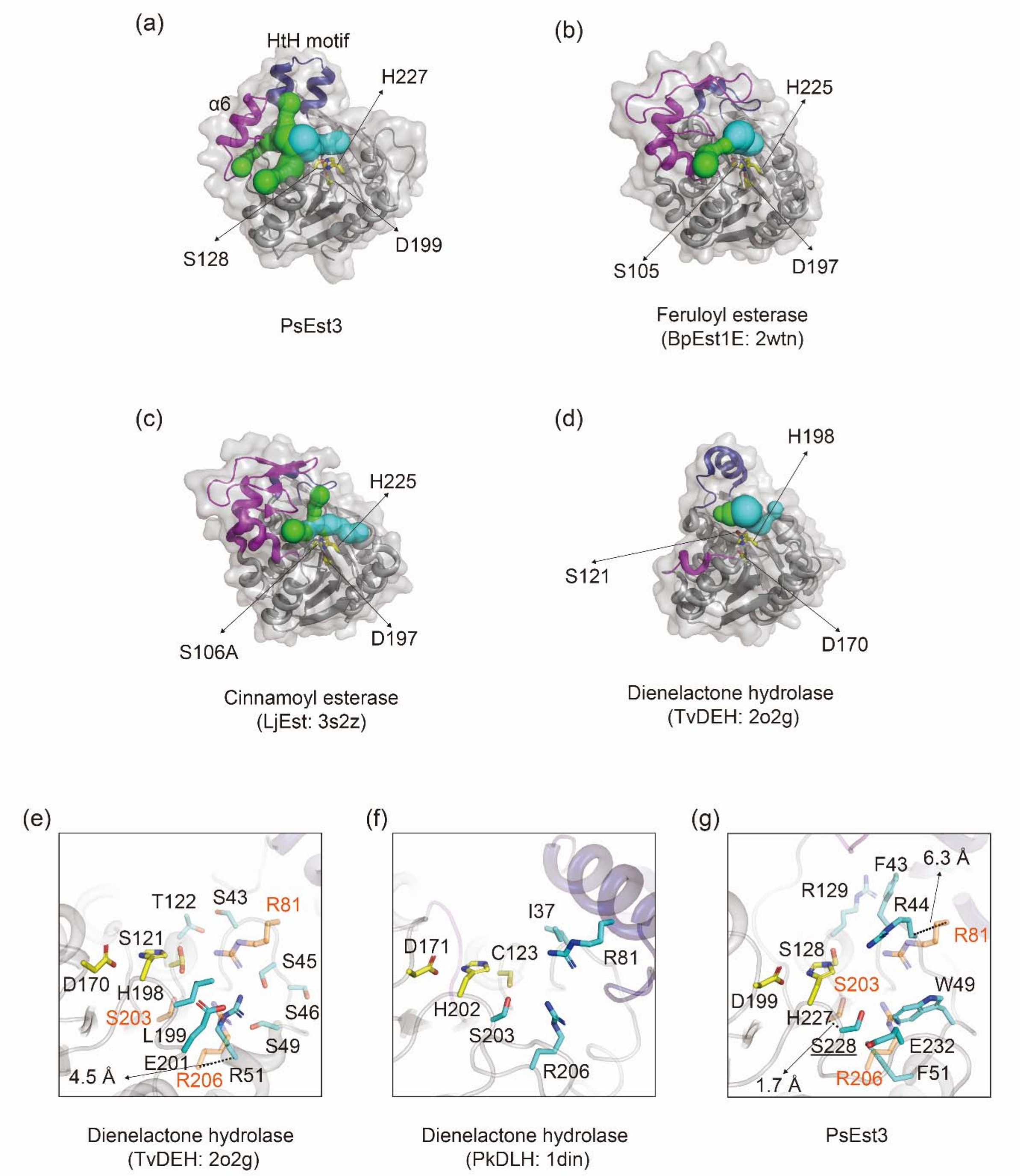

Figure S3 Comparison of PsEst3 with other esterases. (a)–(d) Surface and cartoon representation of PsEst3, feruloyl esterase of *Butyrivibriov proteoclasticus* (BpEst1E, 2wtn), cinnamoyl esterase of *Lactobacillus johnsonii* (LjEst, 3s2z), and dienelactone hydrolase of *Pseudomonas knackmussii* (PkDLH, 1din). Unique structural features of PsEst3 (α6 and HtH motifs) and the corresponding regions of other esterases are highlighted as *magenta* and *dark blue* within the cartoon model. Active sites of esterases are shown as *cyan* spheres, and channels that connect solvent regions and the active sites are represented as *green* spheres. The catalytic triads are represented as *yellow* sticks. (e)–(g) Magnified comparisons of TvDEH, PkDLH and PsEst3 active pockets. The catalytic triad and residues involved

in dienelactone recognition are represented as *yellow* and *blue* sticks, respectively. PsEst3 residues counterpart to those of dienelactone recognition in PkDLH are represented as *cyan* sticks. The structures of TvDEH and PsEst3 are superposed with that of PkDLH. The carboxylate-recognition residues, R81, S203, and R206, of PkDLH are represented as *orange* sticks in the relative orientation. The distance between Cα atoms of the carboxylate-recognition residues of PkDLH and corresponding residues of TvDEH and PsEst3 are designated as *black* dashed line. S228 of PsEst3 is underlined for highlighting complete conservation of S203 of PkDLH.

(a)

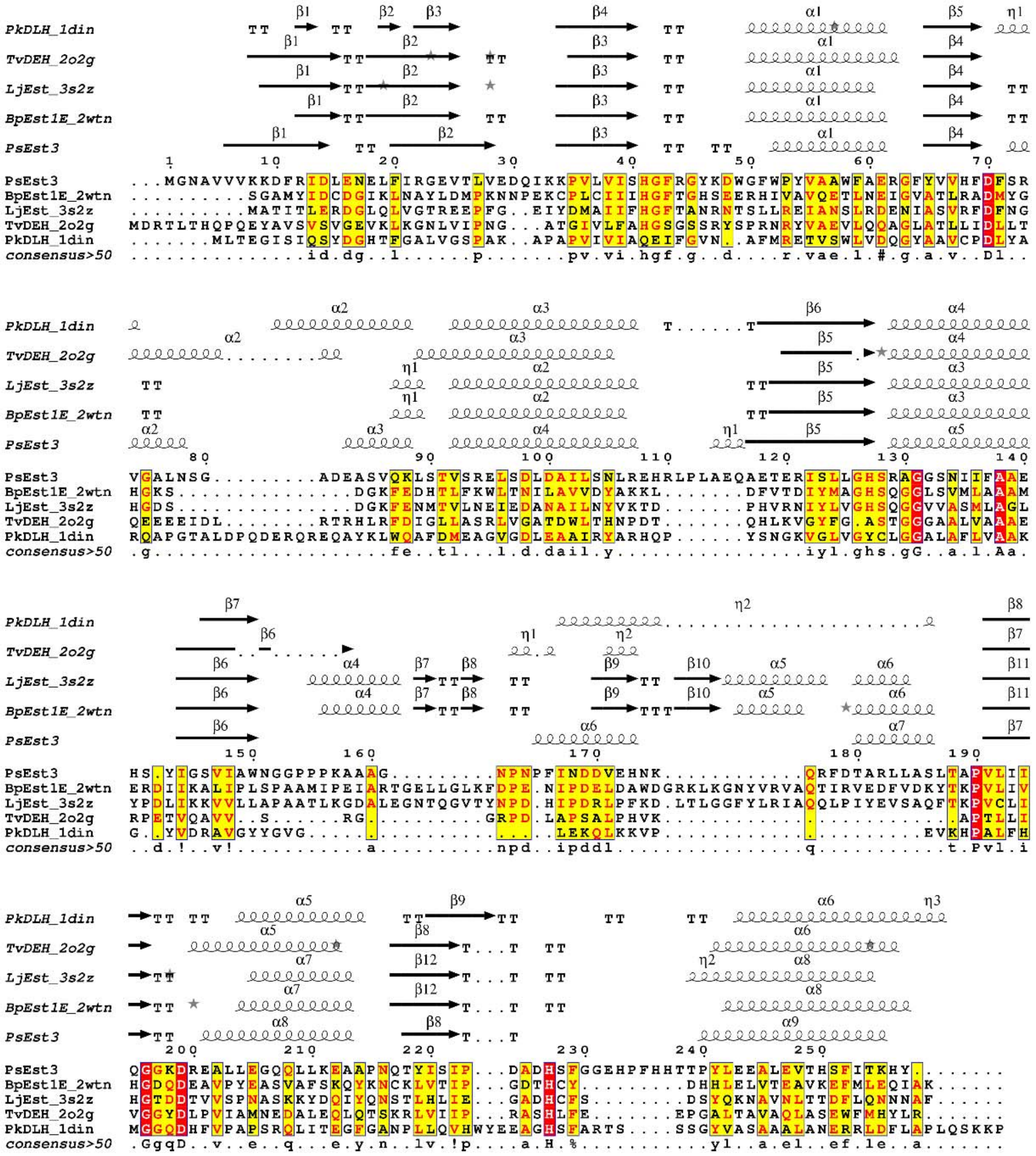
PkDLH_1din
TvDEH_2o2g
LjEst_3s2z
BpEst1E_2wtn
PsEst3
consensus>50

(b)

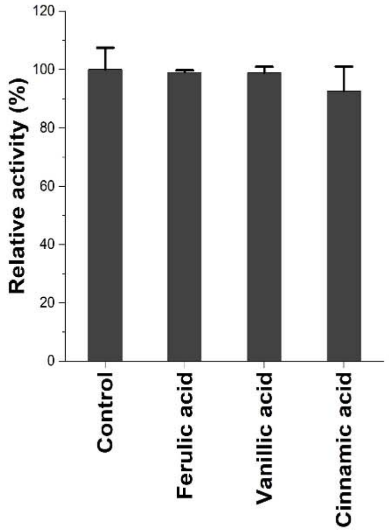
Relative activity (%)
0
20
40
60
80
100
120
Control
Ferulic acid
Vanillic acid
Cinnamic acid

Figure S4 (a) Multiple sequence alignment of PsEst3 with BpEst1E (2wtn), LjEst (3s2z), TvDEH (2o2g), and PkDLH (1din). Secondary structures of PsEst3, BpEst1E, LjEst, TvDEH, and PkDLH are shown in the upper region of the sequence. (b) Substrate competition assay for ferullic acid, vanillic acid, and cinnamoyl acid.

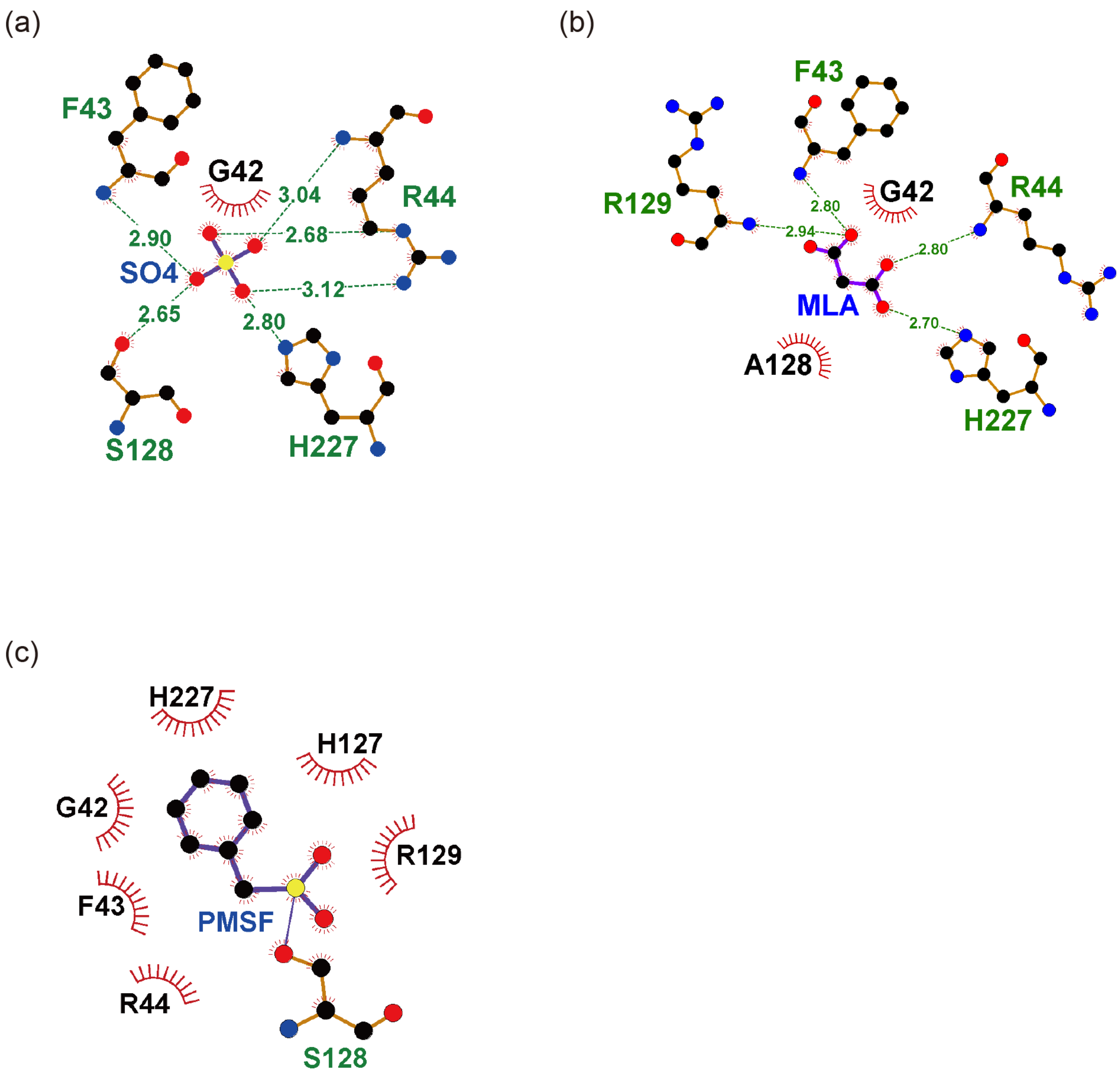

Figure S5 Detailed hydrogen bond interactions between PsEst3 and ligands presented in Fig. 5. (a) Sulfate; (b) malonate; (c) PMSF.
